# Core Hunter: an algorithm for sampling genetic resources based on multiple genetic measures

**DOI:** 10.1186/1471-2105-10-243

**Published:** 2009-08-06

**Authors:** Chris Thachuk, José Crossa, Jorge Franco, Susanne Dreisigacker, Marilyn Warburton, Guy F Davenport

**Affiliations:** 1 Department of Computer Science, University of British Columbia, 2366 Main Mall, Vancouver, BC V6T1Z4, Canada; 2 Crop Research Informatics Laboratory, International Maize and Wheat Improvement Center (CIMMYT), Apdo. Postal 6-641, 06600, México D.F., México; 3 Applied Biotechnology Center, International Maize and Wheat Improvement Center (CIMMYT), Apdo. Postal 6-641, 06600, México D.F., México; 4 International Institute of Tropical Agriculture (IITA), Ibadan, Nigeria; 5 USDA-ARS-CHPRRU, Box 9555, Mississippi State, MS 39762, USA

## Abstract

**Background:**

Existing algorithms and methods for forming diverse core subsets currently address either allele representativeness (breeder's preference) or allele richness (taxonomist's preference). The main objective of this paper is to propose a powerful yet flexible algorithm capable of selecting core subsets that have high average genetic distance between accessions, or rich genetic diversity overall, or a combination of both.

**Results:**

We present Core Hunter, an advanced stochastic local search algorithm for selecting core subsets. Core Hunter is able to find core subsets having more genetic diversity and better average genetic distance than the current state-of-the-art algorithms for all genetic distance and diversity measures we evaluated. Furthermore, Core Hunter can attempt to optimize any number of genetic measures simultaneously, based on the preference of the user. Notably, Core Hunter is able to select significantly smaller core subsets, which retain all unique alleles from a reference collection, than state-of-the-art algorithms.

**Conclusion:**

Core Hunter is a highly effective and flexible tool for sampling genetic resources and establishing core subsets. Our implementation, documentation, and source code for Core Hunter is available at

## Background

**Figure 1:**
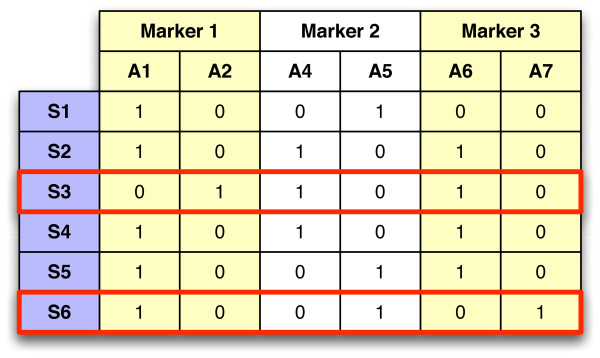
**Example of core subset selection**. A collection of six samples with data collected for three markers, each having two possible alleles, is shown. Each sample shows the presence for one of two possible alleles at each marker position. The two rows bordered in red are a possible core subset which contain all unique alleles found in the original collection.

Genetic resources stored in gene banks are usually sampled with the purpose of evaluating and utilizing them efficiently, as well as studying phenotypic and genotypic diversity, identifying duplicate accessions, and forming core subsets. The aim of the latter activity is to preserve in the sample as much of the diversity present in the original collection as possible. Core subset selection can be based on varying criteria including phenotypic traits or various forms of molecular marker data including, but not limited to, single nucleotide polymorphisms (SNP), amplified fragment length polymorphisms (AFLP), random amplified polymorphic DNA (RAPD), and simple sequence repeats (SSR). A simple example considering SNP data is given in Figure 1. The concept of core collections (or core subsets) was introduced to increase the efficiency of characterizing and utilizing the collections stored in gene banks while preserving the genetic diversity of the collection [1,2].

In many instances, gene bank curators and genetic resource conservation managers need to stratify their sampling procedure prior to forming a core subset. The criteria for stratifying samples may be based on ecogeographical sub-regions or on genetic considerations such as races and/or land-races. Standard stratified sampling strategies seek to maximize the diversity among clusters while minimizing the diversity within groups. Hierarchical clustering algorithms along with statistical models that maximize the probability of assigning accessions into each cluster have been used for this purpose [3-7]. Once clusters are formed, an appropriate allocation method should be used to determine the number of accessions to be drawn from each cluster.

Using phenotypic evaluation and characterization data, the D-method was developed as an allocation criterion for determining the number of accessions to be drawn from each cluster [8]. The D allocation method determines that the size of the sample to be drawn from each cluster should be proportional to the diversity between accessions within that cluster. The authors showed that the D-method produced samples with significantly more diversity than other allocation methods. In another study, the D-method was used, along with other sampling strategies, for forming core subsets of maize using molecular marker data [9]. The results showed that the unweighted pair-group method using arithmetic average clustering (UPGMA) [10] with the D allocation method produced core subsets with significantly more diversity than other methods in terms of genetic distances and diversity indices. Another study, not using the D-method, showed similar results when using deviation sampling with the unweighted pair-group average method for hierarchical clustering [11].

Although the stratified sampling strategy using the D-method proved to be efficient for forming diverse core subsets, these can be formed by a non stratified procedure in which accessions are directly selected from the entire collection by maximizing an objective function. In [12], Schoen and Brown addressed the issue of how to use genetic markers to sample collections of wild related species while maximizing allelic richness. They proposed the M (maximization) strategy that maximizes the number of observed alleles at each marker locus. A study using computer simulation for comparing the retention of neutral alleles when forming core collections using non marker-based random sampling and stratified random sampling strategies versus the M strategy using genetic markers, found the M-strategy very effective for retaining widespread and low frequency alleles [13].

MSTRAT, a local search algorithm based on the M-strategy, has been proposed [14]. As described by the authors, MSTRAT uses a maximum iterative improvement search and consists of (1) forming a subset of *n *accessions chosen at random from the *N *accessions of the whole collection, (2) all possible subsets of size *n *- 1 are tested for allele diversity and the subset showing the highest level of richness is retained, and (3) the accession bringing the greatest increment in the diversity criterion among the remnant accessions is added, forming an new subset of size *n*. Steps (2) and (3) are repeated until the richness of the subset is no longer improved. The diversity of the core subsets formed is measured using a score of allele richness.

The objectives of MSTRAT differ from those of the D-method. It has been suggested that core subsets can be formed that either include rare and localized alleles, which will maximize the total allelic diversity in the core (as favored by taxonomists and geneticists), or can be constructed by including widely adapted accessions that maximize the representativeness of the genetic diversity in the core (which is the breeder's preference) [15]. The objective of the D-method is to select the most diverse accessions in terms of genetic distances among genotypes, whereas the M-strategy emphasizes selecting accessions with the most diverse alleles. This was confirmed in studies, using molecular marker data, which found that MSTRAT is very effective for retaining widespread and low frequency alleles and thus for forming core subsets with high allele richness and a low proportion of non-informative alleles [9,14]. Furthermore, it was found that MSTRAT formed core subsets with more allelic diversity than the D-method in most populations [9]. In general, the D-method formed core subsets having higher average genetic distances between genotypes than MSTRAT.

Recently, Power Core, a new algorithm also based on the M-strategy was proposed [16]. However, Power Core's algorithm differs significantly from existing ones. The authors proposed a deterministic heuristic search, based on the concepts of A* search [17] – an exhaustive graph searching algorithm, guaranteed to find an optimal solution. As suggested by the authors, it would be infeasible to run A* search on even moderately sized problem instances (collections), due to its exhaustive nature. Therefore, the deterministic heuristic they propose is necessary to ensure Power Core terminates in a reasonable amount of time. Although their algorithm is not guaranteed to find optimal solutions, it found core subsets that were superior to those found by MSTRAT, as defined by their proposed evaluation criteria of variable coverage: the ratio of unique values present in the core subset versus those found in the entire collection, averaged over all variables [16].

Other sampling procedures have been proposed such as genetic distance sampling [18], which finds core subsets guaranteeing no two accessions are within a defined distance of each other. In this way, the user need not specify the core size; however, the choice of an appropriate value for the distance parameter can be as problematic as the selection of the core size itself. Another iterative procedure, least distance stepwise sampling (LDSS), was proposed which uses the distances and groupings from hierarchical clustering to determine which accessions to eliminate, and which to add in each step of the procedure until the desired core size has been attained [19]. While both methods employ the use of genetic distances during their heuristic sampling, neither attempts to directly optimize them.

A first objective of this paper is to demonstrate the effectiveness of formally treating core subset selection as an optimization problem. This entails first defining which characteristics of the core subset should be optimized. These may include the average genetic distance between accessions in the core subset, and/or its redundancy of particular alleles, amongst other criteria. Even constraints on the core subset such as ensuring it does not contain two accessions within a threshold distance of each other, as guaranteed by genetic distance sampling, can be treated in a broader view as an optimization problem. We will show that once the criteria for the desired core subset is well defined, the selection can be effectively and efficiently handled by sophisticated search algorithms capable of finding as good or better core subsets when compared with existing methods.

Despite incremental improvements to the challenge of selecting the best core subsets, most existing algorithms and methods currently address either allele representativeness (breeder's concept) or allele richness (taxonomist's perspective). A second objective of this paper is to propose an algorithm capable of selecting core subsets having high average genetic distance between accessions or a rich genetic diversity overall, or a core subset that considers both criteria. A method for combining discrete molecular marker data and continuous genetic data for core subset selection has been previously proposed [20]. We generalize this notion in order to consider any number of optimization criteria simultaneously. In this paper we limit our focus to discrete molecular marker data, although the same approach could be extended to consider continuous genetic data.

We present Core Hunter, an algorithm based on an advanced stochastic local search method. Results from the diversity of the core subsets selected by Core Hunter are compared with the diversity of the core subsets formed using the current state-of-the-art methods with an available implementation: D-Method [8], MSTRAT [14], and Power Core [16]. We demonstrate that Core Hunter finds as good or better core subsets than other methods for all genetic measures evaluated when attempting to optimize a single genetic measure. Furthermore, by attempting simultaneous optimization of multiple genetic measures, Core Hunter often finds core subsets that simultaneously have higher average genetic distance and genetic diversity values than any reported by the other algorithms evaluated. We also demonstrate that Core Hunter is able to find smaller core subsets which maintain all unique alleles found in a reference collection, than all other methods evaluated.

## Methods

To simplify the discussion that follows, we first formalize the core subset selection problem.

### The core subset selection problem

Let *S *denote the original collection of resources and *γ*, 0 ≤ *γ *≤ 1, the sampling intensity used to form the core subset. Furthermore, let **C**(*S*) be the set of all possible core subsets of *S *of size *n*, *n *= *γ*·|*S*|. Finally, let *F *be the objective function we wish to maximize. *F *may be a genetic diversity measure such as Shannon's diversity index, an average genetic distance within a population, possibly measured by Modified Rogers distance, or some multi-objective function which we will detail next. Formally, we wish to select an optimal core subset c*, c* ∈ **C**(*S*), such that *F*(c*) = max{*F*(*c'*)|*c' *∈ **C**(*S*)}.

### The proposed pseudo-index for integrating genetic distances and diversity indices

An advantage of using search algorithms for core subset selection is the potential to select core subsets that attempt to optimize more than one criterion. As an example and given the above definition of core subset selection, let *F*(*c'*) = *α*·*F*_*distance*_(*c'*) + (1 - *α*)*F*_*diversity *_(*c'*), where *α*, 0 ≤ *α *≤ 1, is a weight associated with genetic distance. In this formulation, if *α *= 1.0, then a core subset would be selected which attempts to maximize the average genetic distance, using Modified Roger's distance for example, while genetic diversity, possibly Shannon's diversity index, would not be considered. Likewise, one could attempt to maximize the genetic diversity by setting *α *= 0.0. However, for the case of 0 <*α *< 1, a core subset will be formed by attempting to maximize both genetic distance and diversity proportional to the weight assigned to *α*. This concept is easily generalized to incorporate any *k *measures as shown below, where *F*_*i*_(*c'*) ≥ 0, 1 ≤ *i *≤ *k*, and .

(1)

Indeed, this is a common approach in multi-objective optimization referred to as Pareto optimization [21]. We stress that the pseudo-index does not provide any biological insight into the chosen samples; rather, it serves only as a mean for attempting optimization of more than one genetic measures simultaneously, based on the weights assigned to standard measures. Another common approach in multi-objective optimization is Pareto ranking [22], a technique not explored further here.

### The Core Hunter algorithm for the proposed pseudo-index

The Core Hunter algorithm uses an advanced stochastic local search (SLS) algorithm, replica exchange Monte Carlo [23-25], to maximize the pseudo-index we propose above. This search method has been effectively used to solve high dimensional search problems containing many local maxima embedded in rugged search terrains in many areas of study including spin glasses [26,27] and protein folding [28]. Given the high dimensionality of the core subset selection problem, and the vast number of possible core subsets, this was deemed as a necessary alternative to the use of simpler search methods, such as iterative improvement (hill climbing), which are more likely to become trapped in local maxmima due to their greedy nature. We now provide a brief overview of the algorithm. The reader is referred to a review of extended ensemble Monte Carlo algorithms [25] for further details.

For each replica, Monte Carlo search initiates by randomly selecting *n *accessions from the reference set to form the initial solution, where *n *is the number of accessions desired in the core subset. The search proceeds by perturbing the current solution and exploring the so-called search neighborhood. In this application, our search neighborhood consists of all possible subsets that differ from our current solution, by at most one accession. Thus, to evaluate a new potential solution found in the search neighborhood, we perturb the current core subset by randomly removing an accession and adding a new accession from the original collection. This is commonly referred to as a 1-exchange neighborhood. While it is trivial to extend this concept to a *δ*-exchange neighborhood, *δ *> 1, it was deemed unnecessary given our results. Note that when the core subset need not be of a specific size, it is possible that a perturbation remove an accession, but not add another, or vice versa, in order to shrink or grow the core subset. This is useful, for instance, when attempting to find the smallest core subset which contains all unique alleles of a collection. After perturbing a current solution (core subset) *s *to form an alternate core subset *s'*, the so-called Metropolis criterion is used to determine if the new core subset *s' *should be accepted. The probability of accepting *s' *as the new solution can be expressed as

(2)

where Δ*F *:= *F*(*s'*) - *F*(*s*) is the difference in the pseudo-index score between the new (*s'*) and old (*s*) solutions (core subsets), and *t *denotes the temperature of the replica.

Intuitively, a replica at a higher temperature is more likely to accept a bad transition, one where the new core subset has a worse score than the original. Accepting bad transitions to core subsets with worse scores than a previous solution allows the search to proceed without being trapped at a local maximum. This is a fundamental difference from other search algorithms such as maximum iterative improvement that is implemented in the MSTRAT program [14]. Conversely, replicas at lower temperatures are less likely to accept worsening transitions, and therefore converge towards a solution.

For the duration of the search, *k *independent replicas are maintained, each performing a Monte Carlo search as described above at, a unique temperature *t*_*i*_, 1 ≤ *i *≤ *k*. Replicas are ordered such that *t*_1 _<*t*_2 _<...<*t*_*k*_. Neighboring replicas periodically swap their core subsets in order to allow solutions that have stagnated to break out of local maximums and to allow promising solutions to converge. The probability of swapping the current solutions of replicas *i *and *i *+ 1, namely the core subsets *s*_*i *_and *s*_*i*+1_, can be expressed as

(3)

where *ψ *is the product of the pseudo-index difference and inverse temperature difference:

(4)

As the probability of accepting an exchange of core subsets of two replicas drops exponentially as the temperature difference between them increases, potential replica exchanges are only considered between neighboring temperatures [28].

Therefore, the search consists of performing a Monte Carlo search independently for a number of replicas. In brief, each replica is a potential solution and represents a core subset. After each Monte Carlo search has progressed for a fixed number of steps, replica exchanges are considered, temperatures are possibly swapped, and a Monte Carlo search begins again for each replica. The best solution, among all replicas, is tracked during the entire search. After a fixed runtime, the best solution observed is reported.

### Core Hunter with stratified sampling

As previously stated, it is sometimes necessary to first stratify samples based on ecogeographical information, and other criteria, prior to forming core subsets. In these cases, Core Hunter can be used in a complementary manner with the D-method, or similar allocation methods. After stratification, the D-method will determine the number of resources to sample from each cluster, and Core Hunter can then be run independently on each cluster at the specified sampling intensity.

### Genetic distances and diversity indices

To evaluate the quality of core subsets formed by the different algorithms, and as compared with the original collection, similarly to a previous study [9] we use two genetic distances between genotypes and three diversity indices that can be incorporated into the pseudo-index we propose above. Before providing precise definitions of these measures, we pause to give a brief summary of the purpose of each measure and some insight into how the measures differ from one another. For an in depth treatment of appropriate applications of various genetic measures, and how they relate mathematically, the reader is referred to a review article by Reif *et al*. [29] and references therein, including a review article by Mohammadi and Prasanna [30].

Genetic distances are a measure defined between two samples in order to quantify their degree of dissimilarity; simply put, the larger the value of a genetic distance, the more genetically different the two samples are. Conversely, redundant or highly similar pairs of accessions can easily be identified within a collection as those with very low genetic distance between each other. As a genetic distance measure is defined between a pair of samples, and not an entire collection, it is customary to report the average genetic distance between all unique pairs of samples within a collection. This type of measure is particularly useful for breeders interested in forming core subsets where each chosen accession is sufficiently distant from the others. The two genetic distance measures used here are Modified Rogers (MR) [31] and Cavalli-Sforza and Edwards (CE) [32]. Both measures compare samples at the allelic level. Modified Rogers distance is a refinement of the standard Euclidean distance where each allele is treated as a separate dimension.

Cavalli-Sforza and Edwards distance is similar to Modified Rogers distance, however, it assumes a selective drift model where samples are subject to a low mutation rate and rapid changes in selective pressure [29]. For this reason, Modified Rogers distance may be a more suitable measure than Cavalli-Sforza and Edwards distance in breeding programs where consistent selective pressure is applied for particular traits. In contrast to genetic distance measures, genetic diversity measures do not consider pairs of accessions, rather the allelic composition of the sample as a whole. Genetic diversity measures are particularly useful for ensuring rare alleles, which may confer disease resistance or some other desirable property, are included during core subset formation. For this reason, these measures are well suited for genetic conservation efforts such as seed banks. Although, they can be equally valuable in breeding programs to ensure a large distribution of alleles is maintained. We consider three genetic diversity indices in this study. The first, Shannon's diversity index (SH), is directly related to Shannon's information content measure [33]. The index is defined in such a way that the largest value attainable occurs when each allele is present only once in the entire sample being measured. Generally speaking, it penalizes redundancy at the allelic level, with respect to the entire sample. Therefore, Shannon's diversity index is an appropriate measure when forming core subsets that attempt to retain as many rare alleles as possible, regardless of their co-location within loci (markers). The expected proportion of heterozygous loci (HE) [34] on the other hand, specifically considers diversity within each loci. Intuitively, since each loci contributes equally to the overall value of this measure, core subsets selected using this measure are less likely to be homozygous for a number of different loci than core subsets selected with Shannon's diversity index. The number of effective alleles (NE), by definition, positively correlates with HE and measures the number of alleles within a loci and how evenly alleles are distributed within that loci [34], averaged over all loci in the sample. Thus, both measures are suitable for selecting core subsets which ensure allelic diversity within and across loci.

In each evaluation of a sample, we also report two auxiliary values: the proportion of non-informative alleles (PN) (see [9] and references therein) and allele coverage (CV) [16]. CV is a simple measure which reports the percentage of alleles retained in a core subset compared with the original collection. This measure is particularly suitable in selecting core subsets for the purpose of allele conservation in gene banks and seed banks. For instance, due to time or financial constraints, it may be desirable to select the smallest core subset possible, which retains all unique alleles found within a larger collection (CV = 100%). PN is defined to be the opposite of CV. Thus, maximizing the one will minimize the other.

We now give the precise definition of these measures below where we let *L *be the number of loci (markers), *n*_*l *_be the number of alleles within the *l*^*th *^loci, and *A *be the number of alleles in the collection (). Furthermore, let  denote the relative frequency of allele *a *over the observed frequency of all *A *alleles (note  = 1), let  denote the relative frequency of the *a*^*th *^allele in the *l*^*th *^loci and let  denote the relative frequency of allele *a *within loci *l *for genotype *x*.

1. The Modified Rogers distance (MR) between a pair of genotypes *x*, *y *is .

2. The Cavalli-Sforza and Edwards distance (CE) between a pair of genotypes *x*, *y *is .

3. The Shannon diversity index (SH) of the entire sample is .

4. The expected proportion of heterozygous loci per individual (HE) is .

5. The number of effective alleles (NE) is .

6. Proportion of non-informative alleles in the core subset (PN) is an auxiliary variable measuring the proportion of alleles lost in a core subset compared with the alleles found in the original collection. Specifically, let  be the set of alleles found in the original collection and let  be the set of alleles found in the core subset. Then .

7. Coverage of alleles in the core subset (CV) is an auxiliary variable measuring the percentage of alleles from the collection which are also present in the core subset. Note that CV = (1.0-PN) * 100.

### Criteria for the best core subset

The best core subset has the highest average genetic distance between accessions, the highest allele richness, and the lowest proportion of non-informative alleles (and equivalently, the highest allele coverage). These criteria are in agreement with previous works that suggest core subsets can be formed with the aim of maximizing the total diversity through allele richness and/or maximizing the representativeness of the genetic diversity in the core subset [9,15].

### Data sets

To evaluate the utility of the proposed pseudo-index and the effectiveness of the Core Hunter algorithm, a number of experiments were conducted to compare them to existing strategies. For comparison with MSTRAT and D-Method, the same three molecular marker data sets from a previous study were used [9]. A brief description of the three data sets follows:

• 'bulk data set':

- 275 samples, having 24 markers and 186 total alleles

- obtained by fingerprinting 275 bulks (populations represented by two bulks of 15 genotypes each) of maize landrace populations from the Americas and Europe, using 24 SSR markers with at least one SSR per chromosome and a total of 186 alleles [35]

• 'accession data set':

- 521 samples, having 26 markers and 209 total alleles

- obtained by fingerprinting 521 maize individuals from 25 maize populations using 26 SSR markers with at least one SSR per chromosome and a total of 209 alleles [36].

• 'populations data set':

- 25 samples, having 26 markers and 209 total alleles

- obtained from the 'accession data set' by grouping the individuals of each population, and calculating the allele frequency per population; this data set had a total of 25 populations and 209 alleles [36].

Comparisons with Power Core used the same 'rice SSR' data set as used in the original study on Power Core [16]. The SSR data set contained values for 1000 individual accessions at 18 loci.

### Implementation and hardware

Core Hunter was implemented in Java (version 1.6.0). Experiments were run on our reference Pentium IV 2.4 GHz processor machines, with 1GB main memory and 256 Kb of CPU cache, running SUSE Linux version 10.1.

## Results and Discussion

In the following section, we compare our proposed algorithm for forming core subsets, Core Hunter, with three state-of-the-art methods for which implementations are available: MSTRAT [14], D-Method [8] and Power Core [16]. Core Hunter is evaluated on the same data sets used in recent studies of these algorithms. Results for MSTRAT and D-Method were reported in a previous study [9] where core subsets were selected using a sampling intensity of 20%, a typical choice suggested in the literature [37,38]. For all comparisons with these methods, Core Hunter also used a sampling intensity of 20%. Specifically, 55 samples were chosen for each core subset of the bulk data set, 104 samples for the accession data set, and 5 samples for the population data. We note that in general the choice of sampling intensity, and thus core subset size, for a particular purpose may be based on many independent factors. These factors may include criteria such as diversity, redundancy and possibly financial constraints.

Power Core results were determined by calculating the seven genetic measures used in this study on the core subset it selected for a rice SSR data set. The core subset selected by Power Core was previously published, consisting of 87 accessions, and made available online [16]. When comparing with Power Core, all core subsets selected by Core Hunter also contain 87 accessions, unless otherwise noted. In that study, the authors of Power Core demonstrated the software is capable of selecting smaller core subsets that maintained all unique alleles from the reference collection than other algorithms they compared against. We repeat this experiment with our Core Hunter algorithm.

We show how core subsets can be selected which attempt to optimize multiple genetic measures simultaneously, respective of an assigned weight, using the pseudo-index proposed above. We also explore how core subsets differ under various sampling intensities. As Core Hunter is a randomized algorithm, we also report on the solution quality variance arising from repeated independent simulations.

### Optimizing a single distance or diversity measure

For all data sets, Core Hunter was run with the objective of optimizing each genetic measure independently (Core Hunter (single)). Results reported for each measure are independent of results reported for all other measures. For each genetic measure being optimized for a given data set, 20 independent runs were performed with a maximum search runtime of 5 CPU minutes.

Table 1 compares Core Hunter with MSTRAT and D-Method and lists the mean value of these independents runs for each combination of data set and genetic measure along with previously reported results for MSTRAT and D-Method [9]. When attempting to minimize the proportion of non-informative alleles (PN) and when attempting to maximize the coverage (CV) for the accession data set, Core Hunter matches the performance of MSTRAT with both algorithms finding an optimal solution (0.0 and 100.0, respectively). In every other instance, for every measure, Core Hunter outperforms both MSTRAT and D-Method for the measure being optimized, often by an important margin. Notably, Core Hunter finds core subsets that, on average, improve upon the number of effective alleles for the accession data set by 17.4%, and the average Modified Roger's distance by 17.2% and 13.7% for the population and bulk data sets, respectively. The lowest increases were observed for the Shannon diversity index on each data set, ranging from 0.2% to 2.0% improvement over MSTRAT. Overall, Core Hunter is able to select better cores than MSTRAT and D-Method, with respect to the single genetic measures being optimized.

**Table 1:** Comparison of core subsets selected by MSTRAT, D-Method and Core Hunter

Strategy	MR	CE	SH	HE	NE	PN	CV
	Bulk data set						

Core Hunter (single)†	**0.572**	**0.641**	**4.531**	**0.667**	**3.446**	**0.000**	**100.000**
Core Hunter (multi)‡	0.506	0.598	4.513	0.662	3.403	0.015	98.500
MSTRAT	0.477	0.571	4.493	0.649	3.217	0.021	97.900
D-Method^§^	0.503	0.578	4.411	0.626	2.980	0.066	93.400
COLLECTION	0.440	0.521	4.399	0.620	2.937	0.000	100.000

	Accession data set						

Core Hunter (single)†	**0.694**	**0.752**	**4.670**	**0.676**	**3.501**	**0.000**	**100.000**
Core Hunter (multi)‡	0.659	0.733	4.613	0.650	3.281	0.084	91.600
MSTRAT	0.647	0.718	4.579	0.624	2.982	**0.000**	**100.000**
D-Method^§^	0.653	0.719	4.525	0.619	2.963	0.164	83.600
COLLECTION	0.630	0.696	4.467	0.591	2.742	0.000	100.000

	Population data set						

Core Hunter (single)†	**0.442**	**0.540**	**4.503**	**0.619**	**2.997**	**0.177**	**82.300**
Core Hunter (multi)‡	0.396	0.508	4.482	0.609	2.969	0.225	77.500
MSTRAT	0.357	0.465	4.450	0.593	2.763	0.183	81.700
D-Method^§^	0.377	0.485	4.409	0.579	2.702	0.264	73.600
COLLECTION	0.357	0.455	4.466	0.592	2.749	0.000	100.000

†each selection criteria was attempted to be optimized independently by performing 20 runs with 100% weight given to the respective selection criteria during each run. Results reported for each measure are independent of results reported for all other measures.

‡20 independent runs were performed with equal weight given to each of the seven measures in an attempt to maximize (minimize) all measures simultaneously.

§for each measure, results are shown for the best performing strategy as reported in [9].

Table 2 compares Core Hunter's performance with Power Core. Both algorithms select core subsets having an optimal solution for proportion of non-informative alleles (0.0) and coverage (100.0). For every other measure, Core Hunter outperforms Power Core with respect to the measure being optimized. The least improvement is observed for Shannon's diversity index, with Core Hunter improving upon Power Core by 2%. Core Hunter finds core subsets having values of Modified Roger's and Cavalli-Sforza and Edwards' distance that are 5% better than those of Power Core. The most important gain is for the number of effective alleles, with Core Hunter improving upon Power Core by 21%.

**Table 2:** Comparison of core subsets selected by Power Core and Core Hunter

Strategy	MR	CE	SH	HE	NE	PN	CV
Core Hunter (single)†	**0.926**	**0.926**	**5.259**	**0.873**	**9.431**	**0.000**	**100.000**
Core Hunter (multi)‡	0.884	0.884	5.157	0.841	7.928	**0.000**	**100.000**
Power Core	0.880	0.880	5.131	0.834	7.444	**0.000**	**100.000**
COLLECTION	0.733	0.733	4.397	0.659	3.700	0.000	100.000

†each selection criteria was attempted to be optimized, independently, by performing 20 runs with 100% weight given to the respective selection criteria during each run. Results reported for each measure are independent of results reported for all other measures.

‡20 independent runs were performed with equal weight given to each of the seven measures in an attempt to maximize (minimize) all measures simultaneously.

While Core Hunter is capable of selecting core subsets which meet or exceed the quality of those chosen by existing software for a particular genetic measure, an important question is how the values of the other measures, which are not considered during optimization, were affected. These values are reported in Table S1 and Table S2 [see Additional file 1] and we summarize the findings here. The following general trends were noticed. When Core Hunter only attempts to optimize the auxiliary measures CV and PN, core subsets were selected that in general had worse values than at least one of the other algorithms for every other measure. Conversely, Core Hunter consistently reported the worse score for CV and PN measures when attempting to optimize any other measure. This suggests selecting core subsets which attempt to minimize allele redundancy does not necessarily result in core subsets which have high average genetic distance or diversity, at least in the case of core subsets found by Core Hunter.

Another overall trend that was observed was an apparent trade-off between optimizing genetic distance measures and optimizing genetic diversity measures. This observation was made in a previous study comparing D-Method and MSTRAT [9] and the results for Core Hunter are no exception to this trend. When Core Hunter optimizes a genetic diversity measure, it generally finds core subsets having worse values for distance measures and is outperformed with respect to other software. Likewise, when optimizing a genetic distance, Core Hunter often finds core subsets with worse genetic diversity. There are a few interesting exceptions. For the Accession data set (the largest Maize data set), Core Hunter consistently finds better core subsets with respect to all distance and diversity measures, regardless of which of those measures is being optimized, with only one exception: MSTRAT finds a core subset with a better SH value when Core Hunter is optimizing the MR measure. Also of note is that when Core Hunter is optimizing a genetic distance for the rice data set, it finds core subsets with both good average distance and high diversity, compared with other software.

These results motivate further study in a number of interesting directions. In the next sections, we study these trade-offs in more detail to determine if Core Hunter can be used to find core subsets having both high genetic diversity and high average genetic distance. We also consider the case for trying to find core subsets which have desirable properties for a number of measures, simultaneously.

### Simultaneous distance and diversity optimization

To test how effectively Core Hunter can attempt to optimize genetic distance and genetic diversity measures simultaneously, we conducted the following experiments. For each data set, Core Hunter was run with the objective of optimizing both a genetic distance and a genetic diversity index simultaneously, with respect to a weight assigned to each measure proportional to the pseudo-index parameter *α*. One hundred uniform values were tested in the range [0,1]. For each value of the parameter *α*, 20 independent runs were performed for a duration of 5 CPU minutes, and the mean values of both measures were determined.

**Figure 2:**
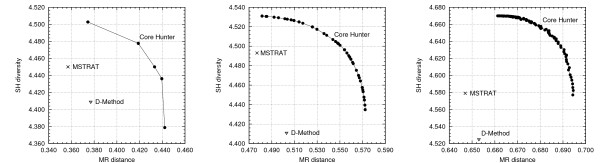
**Maximizing Modified Roger's (MR) distance and Shannon's diversity (SH) index simultaneously**. Core Hunter was run independently for 100 different values of the genetic distance weight parameter, *α*, in an attempt to identify the Pareto frontier for each of the three datasets having results for MSTRAT and D-Method. Each point on the frontier is the mean value of 20 independent runs. Results for the population, bulk and accession datasets are shown on the left, center, and right, respectively.

Figure 2 plots the values of the average Modified Roger's distance versus the Shannon's diversity index values of the core subsets selected by MSTRAT, D-Method, and Core Hunter for different values of *α*. The highest average Modified Roger's distance is observed when all weight is given to genetic distance (i.e., *α *= 1.0). Likewise, the Shannon's diversity index value is highest when no weight is given to genetic distance (i.e., *α *= 0.0). In all other cases (0 <*α *< 1), a trade-off between the measures can be observed, relative to their respective weight in the pseudo-index. This trade-off is characterized as the Pareto set, or Pareto frontier. Note that sufficiently close values of *α* may not necessarily result in distinct core subsets being selected. Therefore, larger data sets (at the same sampling intensity) may result in a larger Pareto set, as is the case when comparing the population data set (Figure 2 (left)) to the accession data set (Figure 2 (right)). Interestingly, in all three data sets, there are values of *α *such that Core Hunter selects core subsets that simultaneously have better genetic distance and genetic diversity than the core subsets selected by either MSTRAT or D-Method.

**Figure 3:**
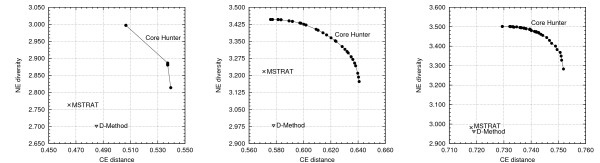
**Attempting to maximize Cavalli-Sforza and Edwards' (CE) distance and the number of effective alleles (NE) simultaneously**. Core Hunter was run independently for 100 different values of the genetic distance weight parameter, *α*, in an attempt to identify the Pareto frontier for each of the three data sets having results for MSTRAT and D-Method. Each point on the frontier is the mean value of 20 independent runs. Results for the population, bulk and accession datasets are shown on the left, center, and right, respectively.

The same experimental protocol was used for simultaneous optimization of the Cavalli-Sforza and Edwards distance and the number of effective alleles diversity index. The margin by which Core Hunter outperforms MSTRAT and D-Method is even larger, with results shown in Figure 3. For the accession data set (Figure 3 (right)), Core Hunter outperforms both algorithms for both measures simultaneously, regardless of the weights assigned to the respective measure.

**Figure 4:**
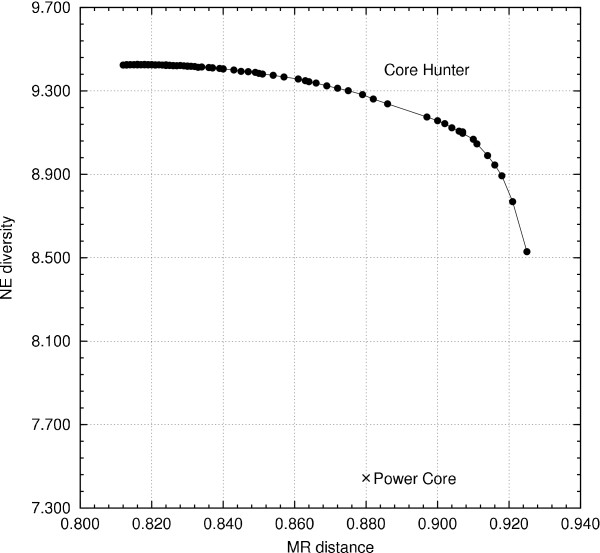
**Attempting to maximize Modified Roger's (MR) distance and the number of effective alleles (NE) simultaneously**. Core Hunter was run independently for 100 different values of the genetic distance weight parameter, *α*, in an attempt to identify the Pareto frontier on the rice SSR data set. Each point on the frontier is the mean value of 20 independent runs.

Figure 4 shows the results of attempting to optimize Modified Roger's distance and the number of effective alleles diversity index compared to the performance of Power Core. Regardless of the value of *α*, Core Hunter always finds core subsets with a higher number of effective alleles. When *α *< 0.88, the core subset selected by Power Core has a higher average Modified Roger's distance than those selected by Core Hunter. However, when more weight is given to genetic distance (i.e., *α *≥ 0.88), Core Hunter easily finds core subsets having better average Modified Roger's distance and a higher number of effective alleles.

As was observed when optimizing a single measure, there is a clear trade-off between genetic distance and genetic diversity. The resulting core subsets discussed above do not have as high average genetic distance than core subsets optimized solely for that property. The same can be said regarding genetic distance. There is a necessary trade-off of one type of property to benefit the other. However, as shown above, core subsets can be selected that still exhibit high average genetic distance and diversity, especially when compared with core subsets formed by MSTRAT, D-Method, or Power Core.

### Optimizing multiple genetic measures

To test the performance of Core Hunter when attempting to optimize more than two measures, the algorithm was run with weights assigned to all seven genetic measures detailed in this paper, for each data set we tested. In all cases, Core Hunter was run for 20 independent trials of 5 CPU minutes.

For the comparison with MSTRAT and D-Method, the mean solution quality is reported in Table 1 (Core Hunter (multi)) with each measure given equal weight. For each of these data sets, Core Hunter is able to select a core subset which better optimizes each genetic measure simultaneously than MSTRAT or D-Method, with the only exception being the proportion of non-informative alleles and coverage. By varying the weights of each measure (i.e., assigning more weight to PN or CV), a core subset can be found which outperforms MSTRAT and D-Method for all measures (data not shown).

Results comparing Core Hunter to Power Core can be found in Table 2 (Core Hunter (multi)). As a goal of Power Core is to optimize coverage, we assigned 99% of the weight to coverage, and distributed the remaining 1% of the weight equally amongst the other measures, for all runs of Core Hunter. Our intention is to show that it is possible to select core subsets which satisfy a primary objective, such as ensuring perfect coverage (CV = 100.0), while still attempting to optimize other measures in the process. Indeed, Core Hunter is able to find a core subset that outperforms Power Core for every genetic measure simultaneously, with the exception of proportion of non-informative alleles and coverage, as both algorithms find an optimal solution.

As discussed in the previous section, the core subsets found when optimizing multiple criteria generally perform worse with respect to individual measures when compared with the core subsets selected for those specific properties. For instance, when optimizing only Modified Roger's distance, Core Hunter finds core subsets which generally have 5% higher average Modified Roger's distance than the core subsets selected by Core Hunter (multi) [see Additional file 1, Table S1]. While these core subsets have higher average Modified Roger's distance, they have 6% less allele coverage on average. Thus, the various trade-offs between genetic distance, diversity and preservation of rare alleles must considered in the context of the intended purpose of the core subset being formed. While optimizing a single one of these criteria will be important in some instances, it will often be the case that finding a suitable balance will be the desired outcome.

### Selecting minimal size core subsets with perfect coverage

With respect to genetic data sets, a goal of Power Core is to select a core subset that retains all unique alleles found in the collection (perfect coverage) and is as small as possible. Using the SSR rice dataset, Kim *et al*. demonstrate that Power Core is able to select smaller core subsets having perfect coverage than a random selection method (R-core), a proportional selection method (P-core) and MSTRAT [16]. Details of the experimental protocol and other selection methods are given in [16].

Core Hunter was run on the same data set, for 20 independent runs of 5 CPU minutes, with the objective of finding the smallest core subset, which retains all unique alleles. Results for Core Hunter and those previously reported in [16] can be found in Table 3. All 20 independent runs of Core Hunter found core subsets having perfect coverage while containing only 80 accessions, 7 accessions fewer than Power Core.

**Table 3:** Comparison of core subset size and coverage

Strategy	Coverage (CV) %	Number of entries
Core Hunter	**100.0**	**80**
Power Core†	**100.0**	87
MSTRAT†	88.9	87
P-core†	55.0	100
R-core†	46.8	100

†results were previously reported in [16].

### Effect of sampling intensity

The choice of sampling intensity when forming core subsets is usually determined by a number of factors. In the previous section the core subset was not intended to be a fixed size, rather the smallest possible size which produced a core subset that retains all unique alleles of a collection. This is beneficial for the application of genetic conservation of rare alleles. Sampling intensity can be chosen based on preliminary estimations of redundancy in the original collection, which is an approach taken in the program MSTRAT [14]. Often, the sampling intensity is chosen based on a combination of factors, including financial constraints, time constraints, and the particular application for which the core subset is intended. A sampling intensity of 5% to 20% has been suggested by many authors in literature [2,12,37,38].

To determine the effect of sampling intensity on the various genetic measures we selected core subsets for the three Maize data sets using two new sampling intensities and compared the results to the core subsets selected using the default 20% sampling intensity. Results for 10% and 30% sampling intensity are shown in Tables S3 and S4 [see Additional file 1] and are summarized here. Two very clear, yet expected, trends were observed. With very few exceptions, core subsets selected with a 10% sampling intensity generally had higher average genetic distance and higher genetic diversity when compared with the core subsets selected using 20% sampling intensity. However, the resulting core subsets did not preserve rare alleles as effectively as the larger core subsets unless the genetic measure being optimized was specifically PN or CV. Core subsets selected with a 30% sampling intensity preserved rare alleles as well or better than smaller core subsets as would be expected. However, the core subsets selected generally had worse average genetic distance and lower genetic diversity. There were exceptions noticed with regards to the Population data set which are probably explained by the small size of the original collection.

Overall, it was observed that a small sampling intensity of 10% results in core subsets which have high average genetic distance and diversity and could be an appropriate choice for breeding programs. The higher sampling intensities selected more homogeneous core subsets that preserved rare alleles better and would be an appropriate choice for applications involving genetic conservation.

### Variability of solution quality

**Figure 5:**
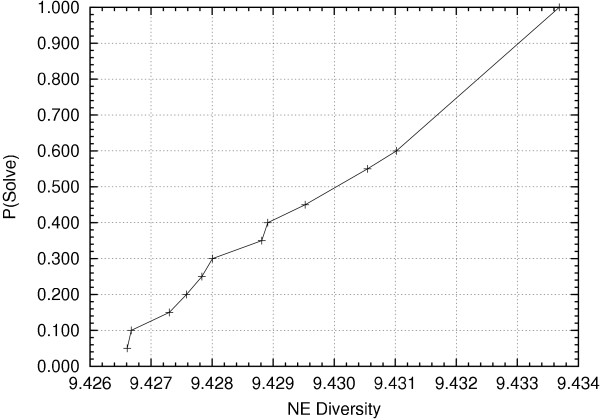
**Solution quality variance**. Cumulative distribution of solution quality, over 20 independent runs, is shown for the rice SSR data set when attempting to maximize the number of effective alleles diversity index.

As Core Hunter is a randomized algorithm, solution quality may vary between independent runs for the same problem instance. However, it was observed that in most cases all independent runs of Core Hunter for a particular combination of data set and genetic measure converge to the same solution quality, yielding negligible variance. The largest range of solution quality values observed involved the optimization of the number of effective alleles (NE) diversity index for the rice SSR data set, [9.42661, 9.43369], having a difference of less than 0.008. The cumulative distribution of solution quality for 20 independent runs is shown in Figure 5. The second largest range of values, also for the rice data set, involved the optimization of the expected proportion of heterozygous loci per individual (HE) diversity index, [0.87333,0.87364], having a difference of less than 0.0004. In nearly every other experiment, the independent trials converged to solutions having the same score.

## Conclusion

We have demonstrated that our proposed algorithm for core subset selection, Core Hunter, has improved upon state-of-the-art selection methodologies in several ways. Results for four distinct genetic data sets show that Core Hunter, when attempting to optimize a single genetic distance or diversity measure, selects core subsets as good as or better than existing algorithms, often by a significant margin. Furthermore, when using the proposed pseudo-index, the algorithm attempts to optimize multiple genetic criteria simultaneously, often finding core subsets that have better values for all genetic measures evaluated, when compared with existing methods. Therefore, it is now possible to select core subsets which satisfy both the breeders' and taxonimists' perspectives, respective of a weight assigned to each genetic measure as specified by the user. Also, our algorithm is agnostic to the choice of genetic measures. New measures can be incorporated without altering the underlying algorithm. We have further demonstrated that Core Hunter is able to select significantly smaller core subsets that retain all unique alleles within a collection, than other algorithms designed for this purpose such as Power Core.

While we believe Core Hunter will significantly improve the process of core subset selection, there are a number of directions in which this approach can be further improved. First, our algorithm currently considers only genetic data. Selection of crop varieties always depend on phenotypic traits and a sole focus on genetic information may bias results due to non-functional genetic variations. Power Core [16], MSTRAT [14] and D-Method [8] all provide support for using phenotypic measures when selecting core subsets. Second, when combining a large number of molecular marker data with phenotypic variables, it is challenging to come up with a unified approach so that information on both data sets can be utilized. While Core Hunter is freely available for use, it currently lacks a rich graphical user interface such as those found in Power Core and MSTRAT. In order to make Core Hunter more accessible to users, development has begun on a rich graphical user interface as well as a web based interface. Announcements regarding these efforts will be made on the project website.

## Authors' contributions

GD, JC, and JF proposed the project, and collaborated with CT on the algorithm development and design and analysis of experiments. CT proposed the algorithm, implemented it, and performed all new experiments. SD, MW provided advice, data and feedback throughout the process. CT and JC wrote the initial manuscript with all authors contributing to the final version.

## Supplementary Material

Additional file 1 **Supplemental results**. Expanded listing of Tables 1 and 2 in addition to results for various sampling intensities. Click here for file

## References

[B1] Frankel OH, Brown AHD, Hoden HW, Williams JT (1984). Plant genetic resources today: a critical appraisal. Crop genetic resources: conservation and evaluation.

[B2] Brown AHD (1989). Core collections: A practical approach to genetic resources management. Genome.

[B3] Franco J, Crossa J, Taba S, Shands H (2003). A multivariate method for classifying cultivars and studying group × environment × trait interaction. Crop Science.

[B4] Franco J, Crossa J (2002). The Modified Location Model for classifying genetic resources. I. Association between Categorial and Continuous Variables. Crop Science.

[B5] Franco J, Crossa J, Taba S, Eberhart SA (2002). The Modified Location Model for classifying genetic resources. II Unrestrictive variance-covariance matrices. Crop Science.

[B6] Franco J, Crossa J, Villaseñor J, Taba S, Eberhart SA (1999). A two-stage, three-way method for classifying genetic resources in multiple environments. Crop Science.

[B7] Franco J, Crossa J, Villaseñnor J, Taba S, Eberhart SA (1998). Classifying genetic resources by categorical and continuous variables. Crop Science.

[B8] Franco J, Crossa J, Taba S, Shands H (2005). A sampling strategy for conserving genetic diversity when forming core subsets. Crop Science.

[B9] Franco J, Crossa J, Warburton ML, Taba S (2006). Sampling strategies for conserving maize diversity when forming core subsets using genetic markers. Crop Science.

[B10] Sokal RR, Michener CD (1958). A statistical method for evaluating systematic relationships. University of Kansas Science Bulletin.

[B11] Li CT, Shi CH, Wu JG, Xu HM, Zhang HZ, Ren YL (2004). Methods of developing core collections based on the predicted genotypic value of rice (Oryza sativa L.). Theoretical Applied Genetics.

[B12] Schoen DJ, Brown AHD (1993). Conservation of allelic richness in wild crop relatives is aided by assessment of genetic markers. Proceedings of the Natial Academy of Sciences, USA.

[B13] Bataillon TL, David JL, Schoen DJ (1996). Neutral genetic markers and conservation genetics: simulated germplasm collections. Genetics.

[B14] Gouesnard B, Bataillon TM, Decoux G, Rozale C, Schoen DJ, David JL (2001). MSTRAT: An algorithm for building germplasm core collections by maximizing allelic or phenotypic richness. Journal of Heredity.

[B15] Marita JM, Rodríguez JM, Nienhuis J (2000). Development of an algorithm indentifying maximally diverse core collections. Genetic Resources and Crop Evolution.

[B16] Kim KW, Chung HK, Cho GT, Ma KH, Chandrabalan D, Gwag JG, Kim TS, Cho EG, Park YJ (2007). PowerCore: A program applying the advanced M strategy with a heuristic search for establishing core sets. Bioinformatics.

[B17] Hart PE, Nilsson NJ, Raphael B (1968). A Formal Basis for the Heuristic Determination of Minimum Cost Paths. IEEE Transactions on Systems Science and Cybernetics.

[B18] Jansen J, van Hintum T (2007). Genetic distance sampling: a novel sampling method for obtaining core collections using genetic distances with an application to cultivated lettuce. Theoretical Applied Genetics.

[B19] Wang JC, Hu J, Xu HM, Zhang S (2007). A strategy on constructing core collections by least distance stepwise sampling. Theoretical Applied Genetics.

[B20] Wang J, Hu J, Liu N, Xu H, Zhang S (2006). Investigation of combining plant genotypic values and molecular marker information for constructing core subsets. Journal of Integrative Plant Biology.

[B21] Steuer RE (1985). Multiple Criteria Optimization: Theory, Computation and Application.

[B22] Coello Coello C, Lamont G, Van Veldhuizen D (2007). Evolutionary Algorithms for Solving Multi-objective Problems, Springer.

[B23] Geyer C (1991). Markov chain Monte Carlo maximum likelihood. Computing Science and Statistics: Proceedings of the 23rd Symposium on the Interface.

[B24] Kimura K, Taki K (1991). Time-homogeneous parallel annealing algorithm. Proceedings of the 13th IMACS World Congress on Computation and Applied Mathematics (IMACS'91).

[B25] Iba Y (2001). Extended Ensemble Monte Carlo. International Journal of Modern Physics C.

[B26] Hukushima K, Nemoto K (1996). Exchange Monte Carlo Method and Application to Spin Glass Simulations. Journal of the Physical Society of Japan.

[B27] Hukushima K, Takayama H, Yoshino H (1998). Exchange Monte Carlo Dynamics in the SK Model. Journal of the Physical Society of Japan.

[B28] Thachuk C, Shmygelska A, Hoos HH (2007). A replica exchange Monte Carlo algorithm for protein folding in the HP model. BMC Bioinformatics.

[B29] Reif JC, Melchinger AE, Frisch M (2005). Genetical and Mathematical Properties of Similarity and Dissimilarity Coefficients Applied in Plant Breeding and Seed Bank Management. Crop Science.

[B30] Mohammadi SA, Prasanna BM (2003). Analysis of Genetic Diversity in Crop Plants-Salient Statistical Tools and Considerations. Crop Sci.

[B31] Wright S (1978). Evolution and the Genetics of Populations: A treatise in four volumes.

[B32] Cavalli-Sforza L, Edwards A (1967). Phylogenetic analysis. Models and estimation procedures. American Journal of Human Genetics.

[B33] Shannon C (1948). A mathematical theory of communication. Bell Systems Technical Journal.

[B34] Berg EE, Hamrick JL (1997). Quantification of genetic diversity at allozyme loci. Canadian Journal of Forest Research.

[B35] Dubreuil P, Warburton M, Chastanet M, Hoisington D, Charcosset A (2006). More on the introduction of temperate maize into Europe: Large-scale bulk SSR genotyping and new historical evidence. Maydica.

[B36] Warburton M, Crossa J, Diaz L, Gomez A, Taba S (2004). Diversidad genética en criollos de mais medida por microsatélites. V Congreso Nacional de Biotecnología Agropecuaria y Forestal, Chapingo, México.

[B37] van Hintum TJL, Johnson RC, Hodgkin T (1999). The general methodology for creating a core collection. Core collections for today and tomorrow.

[B38] van Hintum TJL, Brown AHD, Spillane C, Hodgkin T (2000). Core collections of plant genetic resources.

