# Supplementary material for: Core Hunter: an algorithm for sampling genetic resources based on multiple genetic measures
Source: BMC Bioinformatics. 2009 Aug 6;10:243. doi: 10.1186/1471-2105-10-243 (PMC2734557; doi:10.1186/1471-2105-10-243)
Supplement: Additional file 1 — Supplemental results. Expanded listing of Tables 1 and 2 in addition to results for various sampling intensities. [file 1471-2105-10-243-S1.pdf]

Supplemental Material

for

Core Hunter: an algorithm for sampling genetic resources based on multiple genetic measures

Table S1 - Expanded results for Table 1

An expanded version of Table 1 is shown below. Values for all measures are shown for each genetic measure being optimized which is indicated in parenthesis. For instance, Core Hunter (MR) indicates 100% weight was allocated to the MR (Modified Rogers distance) measure. Values reported are the mean of 20 independent runs. Shaded cells indicate the value for Core Hunter was worse than either MSTRAT, D-Method, or both, according to the legend below. Values shown in bold are the best observed overall.

Strategy	MR	CE	SH	HE	NE	PN	CV
	Bulk data set						
Core Hunter (MR)	**0.572**	0.637	4.427	0.639	3.073	0.091	90.900
Core Hunter (CE)	0.569	**0.641**	4.452	0.646	3.145	0.065	93.500
Core Hunter (SH)	0.481	0.578	**4.531**	0.664	3.376	0.027	97.300
Core Hunter (HE)	0.499	0.589	4.520	**0.667**	3.370	0.034	96.600
Core Hunter (NE)	0.478	0.576	4.509	0.660	**3.446**	0.022	97.800
Core Hunter (PN/CV)	0.441	0.524	4.401	0.619	2.950	**0.000**	**100.000**
Core Hunter (multi)	0.506	0.598	4.513	0.662	3.403	0.015	98.500
	Accession data set						
Core Hunter (MR)	**0.694**	0.747	4.571	0.640	3.078	0.148	85.200
Core Hunter (CE)	0.688	**0.752**	4.623	0.660	3.248	0.115	88.500
Core Hunter (SH)	0.662	0.738	**4.670**	0.669	3.349	0.060	94.000
Core Hunter (HE)	0.662	0.738	4.649	**0.676**	3.353	0.116	88.400
Core Hunter (NE)	0.654	0.730	4.622	0.655	**3.501**	0.102	89.800
Core Hunter (PN/CV)	0.637	0.706	4.517	0.605	2.836	**0.000**	**100.000**
Core Hunter (multi)	0.659	0.733	4.613	0.650	3.281	0.084	91.600
	Population data set						
Core Hunter (MR)	**0.442**	0.534	4.379	0.574	2.744	0.335	66.500
Core Hunter (CE)	0.433	**0.540**	4.420	0.591	2.754	0.306	69.400
Core Hunter (SH)	0.374	0.501	**4.503**	0.610	2.916	0.191	80.900
Core Hunter (HE)	0.419	0.524	4.478	**0.619**	2.861	0.254	74.600
Core Hunter (NE)	0.394	0.507	4.472	0.604	**2.997**	0.249	75.100
Core Hunter (PN/CV)	0.360	0.467	4.451	0.592	2.776	**0.177**	**82.300**
Core Hunter (multi)	0.396	0.508	4.482	0.609	2.969	0.225	77.500

* Worse than MSTRAT (blue) Worse than D-Method (green) Worse than both (yellow)

Table S2 - Expanded results for Table 2

An expanded version of Table 2 is shown below. Values for all measures are shown for each genetic measure being optimized which is indicated in parenthesis. For instance, Core Hunter (MR) indicates 100% weight was allocated to the MR (Modified Rogers distance) measure. Values reported are the mean of 20 independent runs. Shaded cells indicate the value for Core Hunter was worse than Power Core. Values shown in bold are the best observed overall.

Strategy	MR	CE	SH	HE	NE	PN	CV
Core Hunter (MR)	**0.926**	**0.926**	5.158	0.857	8.111	0.124	87.600
Core Hunter (CE)	**0.926**	**0.926**	5.158	0.857	8.111	0.124	87.600
Core Hunter (SH)	0.814	0.814	**5.259**	0.870	9.061	0.081	91.900
Core Hunter (HE)	0.777	0.777	5.231	**0.873**	8.958	0.144	85.600
Core Hunter (NE)	0.814	0.814	5.224	0.867	**9.431**	0.108	89.200
Core Hunter (PN)	0.865	0.865	5.065	0.816	6.851	**0.000**	**100.000**
Core Hunter (multi)	0.884	0.884	5.157	0.841	7.928	**0.000**	**100.000**

* Worse than Power Core

Table S3 - 10% Sampling Intensity

The experiments which produced Table S1 were rerun for Core Hunter with a core subset sampling intensity of 10%. Values which are worse than those found with a 20% sampling intensity have been highlighted.

Strategy	MR	CE	SH	HE	NE	PN	CV
	Bulk data set						
Core Hunter (MR)	0.611	0.666	4.404	0.636	3.011	0.167	83.300
Core Hunter (CE)	0.603	0.670	4.436	0.643	3.141	0.129	87.100
Core Hunter (SH)	0.484	0.589	4.559	0.671	3.467	0.032	96.800
Core Hunter (HE)	0.516	0.610	4.541	0.678	3.478	0.086	91.400
Core Hunter (NE)	0.479	0.583	4.534	0.669	3.593	0.065	93.500
Core Hunter (PN)	0.438	0.528	4.419	0.621	2.987	0.000	100.000
	Accession data set						
Core Hunter (MR)	0.709	0.761	4.584	0.653	3.182	0.228	77.200
Core Hunter (CE)	0.701	0.767	4.649	0.674	3.397	0.166	83.400
Core Hunter (SH)	0.677	0.752	4.708	0.682	3.467	0.106	89.400
Core Hunter (HE)	0.663	0.741	4.675	0.693	3.535	0.190	81.000
Core Hunter (NE)	0.663	0.734	4.629	0.663	3.720	0.191	80.900
Core Hunter (PN)	0.643	0.719	4.576	0.619	2.961	0.000	100.000
	Population data set						
Core Hunter (MR)	0.502	0.585	4.258	0.546	2.438	0.512	48.800
Core Hunter (CE)	0.502	0.585	4.258	0.546	2.438	0.512	48.800
Core Hunter (SH)	0.378	0.532	4.420	0.594	2.844	0.368	63.200
Core Hunter (HE)	0.378	0.532	4.420	0.594	2.844	0.368	63.200
Core Hunter (NE)	0.390	0.534	4.404	0.590	2.850	0.383	61.700
Core Hunter (PN)	0.339	0.448	4.364	0.572	2.593	0.349	65.100

* Worse than Core Hunter (20% sampling intensity)

Table S4 - 30% Sampling Intensity

The experiments which produced Table S1 were rerun for Core Hunter with a core subset sampling intensity of 30%. Values which are worse than those found with a 20% sampling intensity have been highlighted.

Strategy	MR	CE	SH	HE	NE	PN	CV
	Bulk data set						
Core Hunter (MR)	0.546	0.615	4.427	0.637	3.035	0.042	95.800
Core Hunter (CE)	0.543	0.618	4.448	0.642	3.114	0.037	96.300
Core Hunter (SH)	0.475	0.569	4.511	0.657	3.307	0.016	98.400
Core Hunter (HE)	0.481	0.571	4.502	0.659	3.302	0.028	97.200
Core Hunter (NE)	0.472	0.567	4.496	0.654	3.361	0.016	98.400
Core Hunter (PN)	0.442	0.523	4.398	0.619	2.927	0.000	100.000
	Accession data set						
Core Hunter (MR)	0.683	0.737	4.554	0.631	3.010	0.103	89.700
Core Hunter (CE)	0.677	0.742	4.596	0.648	3.146	0.087	91.300
Core Hunter (SH)	0.655	0.731	4.636	0.656	3.236	0.037	96.300
Core Hunter (HE)	0.656	0.731	4.616	0.663	3.217	0.081	91.900
Core Hunter (NE)	0.653	0.726	4.599	0.645	3.356	0.074	92.600
Core Hunter (PN)	0.635	0.702	4.494	0.599	2.782	0.000	100.000
	Population data set						
Core Hunter (MR)	0.430	0.522	4.412	0.582	2.745	0.263	73.700
Core Hunter (CE)	0.422	0.525	4.446	0.593	2.829	0.215	78.500
Core Hunter (SH)	0.372	0.484	4.504	0.614	2.887	0.144	85.600
Core Hunter (HE)	0.389	0.493	4.494	0.619	2.856	0.182	81.800
Core Hunter (NE)	0.381	0.493	4.497	0.608	2.965	0.158	84.200
Core Hunter (PN)	0.355	0.466	4.474	0.598	2.760	0.124	87.600

* Worse than Core Hunter (20% sampling intensity)
